# Quality of life of pediatric and adult individuals with osteogenesis imperfecta: a meta-analysis

**DOI:** 10.1186/s13023-023-02728-z

**Published:** 2023-05-24

**Authors:** Susanne Wehrli, Marianne Rohrbach, Markus Andreas Landolt

**Affiliations:** 1 Department of Psychosomatics and Psychiatry, University Children’s Hospital, University of Zurich, Zurich, Switzerland; 2 Division of Child and Adolescent Health Psychology, Department of Psychology, University of Zurich, Zurich, Switzerland; 3 Children’s Research Centre, University Children’s Hospital Zurich, University of Zurich, Zurich, Switzerland; 4 Division of Metabolism, University Children’s Hospital Zurich, University of Zurich, Zurich, Switzerland; 5 University Research Priority Program “ITINERARE –Innovative Therapies in Rare Diseases”, University of Zurich, Zurich, Switzerland

**Keywords:** Osteogenesis imperfecta, Rare disease, Quality of life, Pediatrics, Adult, Physical health, Mental health, PedsQL™, SF-36, SF-12

## Abstract

**Background:**

Osteogenesis imperfecta (OI) is a group of rare inheritable disorders of connective tissue. The cardinal manifestations of OI are low bone mass and reduced bone mineral strength, leading to increased bone fragility and deformity that may lead to significant impairment in daily life. The phenotypic manifestations show a broad range of severity, ranging from mild or moderate to severe and lethal. The here presented meta-analysis aimed to analyze existing findings on quality of life (QoL) in children and adults with OI.

**Methods:**

Nine databases were searched with predefined key words. The selection process was executed by two independent reviewers and was based on predetermined exclusion and inclusion criteria. The quality of each study was assessed using a risk of bias tool. Effect sizes were calculated as standardized mean differences. Between-study heterogeneity was calculated with the I^2^ statistic.

**Results:**

Among the studies included two featured children and adolescents (*N* = 189), and four adults (*N* = 760). Children with OI had significantly lower QoL on the Pediatric quality of life inventory (PedsQL) with regards to the total score, emotional, school, and social functioning compared to controls and norms. The data was not sufficient to calculate differences regarding OI-subtypes. In the adult sample assessed with Short Form Health Survey Questionnaire, 12 (SF-12) and 36 items (SF-36), all OI types showed significantly lower QoL levels across all physical component subscales compared to norms. The same pattern was found for the mental component subscales namely vitality, social functioning, and emotional role functioning. The mental health subscale was significantly lower for OI type I, but not for type III and IV. All of the included studies exhibited a low risk of bias.

**Conclusions:**

QoL was significantly lower in children and adults with OI compared to norms and controls. Studies in adults comparing OI subtypes showed that the clinical severity of the phenotype is not related to worse mental health QoL. Future research is needed to examine QoL in children and adolescents in more sophisticated ways and to better understand the association between clinical severity of an OI-phenotype/severity and mental health in adults.

**Supplementary Information:**

The online version contains supplementary material available at 10.1186/s13023-023-02728-z.

## Introduction

Osteogenesis imperfecta (OI), also known as brittle bone disease, is a rare and heritable connective tissue disorder that has many faces, as it is genetically and phenotypically heterogenous. Historically, OI was considered to be an autosomal dominant disorder caused by a defect in type I collagen. More than 80% of clinical cases are caused by mutations in the COL1A1 and COL1A2 genes causing structural or quantitative alterations of type I collagen [54]. In the last 15 years advances in molecular diagnoses have led to the discovery of at least 20 additional genetic defects leading to OI with autosomal dominant, autosomal recessive and X-linked inheritance. Most of the genes encode proteins involved in collagen synthesis, posttranslational modification, processing, secretion, and maturation but also in general bone mineralization and osteoblast development.

The precise incidence of OI is unknown, but has been estimated to be in the range of 1:15,000–1:20,000 [55, 73]. As the different pathogenic mutations of the genes encoding type I collagen are highly diverse, the phenotypical representation of OI varies as well. Most individuals are categorized with a classification system, which incorporates a mild form (type I), a neonatal lethal type (type II), one that leads to severe deformations (type III), a moderately deforming type (type IV) and lastly, a calcification in interosseous membranes (type V) [22, 95]. Thus, the severity of OI can differ immensely, with some individuals dying before birth (type II), to some almost experiencing no symptoms at all (type I). Two possible and significant consequences of OI are short stature and impaired ambulation often leading to significantly reduced quality of life (QoL) and high morbidity.

To date, there is no cure for OI. However, its symptoms can be managed by administering biophosphonate drugs, physical therapy, and surgery [31, 68]. Whereas intramedullar rods is mainly used to treat OI fractures. Previous findings have shown that individuals with OI have limited mobility and face barriers in various areas of life such as employment and sports, and that coping with these daily realities is challenging and may lead to mental health problems [18]. Compared to population norms, individuals with OI reported higher levels of anxiety, depression, and lower general mental health scores [90]. In a qualitative study with a substantially smaller sample, OI patients attributed their elevated anxiety scores to needle phobias and fear of fractures during certain activities and in busy areas [39]. In addition, children with OI reported feeling lonely because they are socially isolated and judged by their peers based on their appearance [23, 25].

Another outcome that can be negatively affected in individuals with OI is QoL. QoL is a multidimensional concept that includes wide-ranging constructs such as functional status, emotional functioning, health perceptions, and social functioning [16]. Definitions related to QoL vary in the literature, as there are no uniform definitions of which dimensions should be considered when measuring it. However, there is consensus in the literature that QoL should be reported directly by the patient and that it is a multidimensional construct [72]. Nowadays, there are several validated and standardized self-assessments for QoL for both children and adults. Measuring QoL in OI is essential because it captures an individuals’ personal experience and therefore is vital for the successful implementation of interventions and the assessment of novel treatments. Since the various OI subtypes differ greatly in terms of their severity and symptomatology, certain aspects of QoL may be affected stronger than others. For example, previous studies found higher levels of physical burden, but only slightly lower levels of mental health across different OI types compared to healthy controls and norm populations [27, 32, 33, 104]. However, these effects concerning QoL vary among different OI types [32, 33, 87, 97]. To date, no quantitative summary has produced an overview of current findings in the literature. The present meta-analysis aimed to provide such an overview, with the superordinate goal of doing justice to the versatile nature of QoL in the context of OI, for both children and adults. Of course, there are many factors that can influence quality of life, such as social or interindividual factors, but this was not the aim of the present work. We aimed to investigate if and how individuals with OI differ from healthy controls and norms regarding QoL, and if and how QoL differs among different OI subtypes.

## Methods

### Data sources and search strategy

**Fig. 1:**
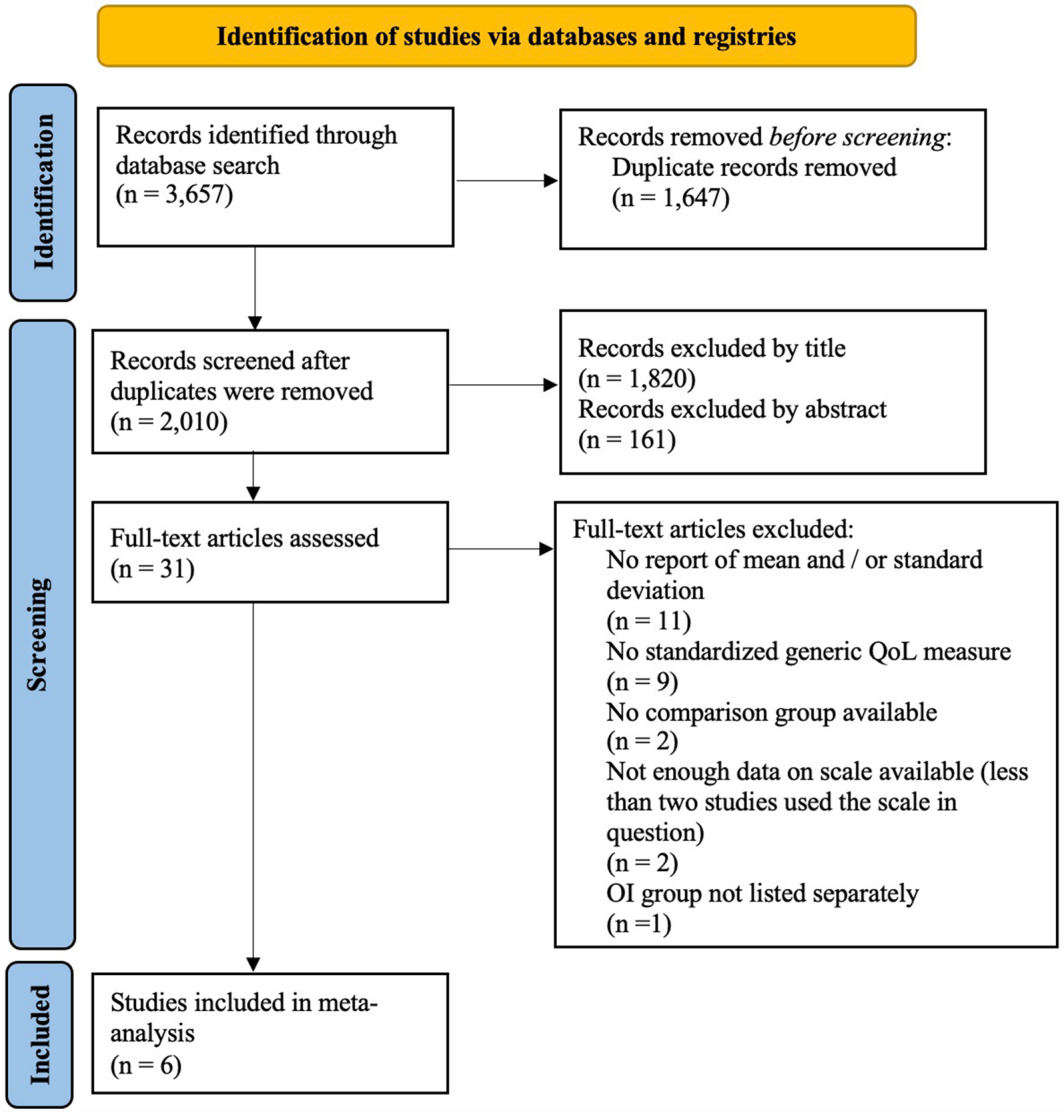
PRISMA flow diagram of study selection

This meta-analysis was pre-registered on PROSPERO (CRD42021276216). The materials and the data are publicly available via the Open Science Framework: https://osf.io/a4dxb/?view_only=efbaa104c7c84a55b639c665059912e1. The initial systematic search was conducted on the 27th of July 2021 by the first author and is illustrated in Fig. 1. A search of psychological and medical databases was conducted including MEDLINE (via PubMed), PsycInfo (via EBSCOhost), CINAHL (via EBSCOhost), PSYNDEX (via EBSCOhost), EMBASE, ProQuest, Dissonline.de, Cochrane Central Register of Controlled Trials (CENTRAL) via Cochrane Library and Clinicaltrials.gov. The search terms were selected by reviewing other meta-analyses and review articles in OI. Search-terms were divided into two groups. The first group consisted of different disease specific names (osteogenesis imperfecta and brittle bone disease). The second group consisted of different terms used to describe QoL (quality of life, health related quality of life, QoL, HRQoL, life quality, health Status and well-being). These two search groups were connected by the Boolean operator “AND”, whereas terms within the groups were connected with the Boolean operator “OR”. The initial search comprised of screening titles and abstracts and was followed by the screening of full-text articles. Following the initial search two additional search strategies were applied to minimize publication bias. First, authors of the included studies were contacted, asking them about unpublished data. Secondly the reference lists from included studies were searched for relevant articles.

### Study selection process

To find eligible studies, a rating scheme was created which consisted of pre-established inclusion and exclusion criteria. The inclusion criteria were as follows: (1) both published and unpublished studies were allowed; (2) language of publication had to be either English or German; (3) the reported data had to be quantitative; (4) the patient population had to be diagnosed with OI; (5a) intervention studies were included if pre-intervention data on QoL was reported; (5b) a comparison group that consisted of either healthy controls, a norm population or OI subgroup had to be included; (6) both self-report or proxy reports were allowed; (7a) QoL had to be assessed with a generic or, (7b) OI-disease specific standardized measure, (8) and lastly authors had to provide means and standard deviations of QoL measures. If the last requirement was not met, but all other inclusion criteria, authors were contacted to provide means and standard deviations.

Exclusion criteria consisted of the following: (1) conference proceedings were not allowed; (2) language of publication was neither English nor German; (3) the reported data was qualitative; (4) the patient population was not diagnosed with OI; (5a) intervention studies were excluded if pre-intervention data on QoL was not reported; (5b) case studies were excluded; (5c) no comparison group that consists of either healthy controls, a norm population or OI subgroups also lead to exclusion; (6a) no standardized assessment of QoL by means of a generic or, (6b) OI-disease specific measure, (6c) usage of a body part specific QoL measure lead to exclusion as well; (7) means and standard deviation of QoL measures not provided.

The rating process consisted of two steps. Firstly, the articles whose full texts were screened, were checked with regards to inclusion and exclusion criteria independently by the first and the last author. Disagreements were resolved through discussion until consensus was reached. Cohen’s Kappa was moderate (κ = 0.66, *p* < 0.001) [58].

### Data extraction

Data from the included studies was independently extracted by the first author and controlled by the last author. Data to be extracted included the following: list of authors, year of publication, country of origin, age group, mean age, comparison group(s), name of QoL instrument, total number of participants, number of participants in the comparison group, number of female, and male participants, QoL mean and standard deviation for OI group, OI subtypes, and lastly information about study quality.

### Quality assessment

The quality of each included study was assessed using a risk of bias tool adapted from the Prevalence Critical Appraisal Tool by Moola et al. [63] (see Additional file 1). The tool consisted of six questions assessing the following: clear definition of inclusion and exclusion criteria; quality and representativeness of the sample for patient and comparison group; validity and reliability of QoL measure; comparability of outcome measure between patient and comparison group; appropriateness of statistical analyses. Each study was rated based on a 4-level response scale (risk of bias: 0 = high, 1 = some concerns, 2 = low, ? = no information) and received a total risk of bias score (0–3 = high risk of bias, 4–7 = some concerns, 8–12 = low risk of bias). All studies were rated by the first author and checked by the last author. All discrepancies between the two were discussed and resolved. The rating of each study can be found in a summary bar plot, illustrating the proportion of studies with a certain risk (Additional file 2).

### Statistical analysis

All analyses were conducted in R [78] with the *metaphor* package [99] and the *dmetar* package [35]. Results were visualized with forest plots. Differences concerning mean levels of QoL-scales were calculated using the standardized mean difference (SMD) and 95% confidence intervals (CIs) using the restricted maximum likelihood estimator (REML) method. REML was selected because it is robust with regard to the calculation of SMDs [38]. Minimal clinically important differences (MCIDs) are not well established for any of the QoL measures used in this study. Therefore, instead of MCIDs the standard rules of thumb were applied to SMDs [83] (i.e., d(0.01) = very small, d(0.2) = small, d(0.5) = medium, d(0.8) = large, d(1.2) = very large, and d(2.0) = huge). Between-study heterogeneity was calculated with the I^2^ statistic. The I^2^ statistic was interpreted with values around 50% or lower being considered as low heterogeneity, whereas values between 50 and 75% were considered to point towards moderate heterogeneity and values above 75% indicated as a high level of heterogeneity [37]. Because the I^2^ statistic depends on the precision of the studies included, prediction intervals were calculated as well [14, 42]. If the I^2^ statistic indicated low levels of heterogeneity, fixed effect models were used, whereas when heterogeneity was moderate, or high, a random effect model was used. Lastly, due to the limited number of studies publication bias was not visually and statistically inspected using a funnel plot [36]. For the Eggers bias test a minimum sample of six studies has been recommended and hence, it was not calculated either [50].

Two of the included papers [44, 46] did not list participants’ mean age, however, they did feature frequency tables which contained age ranges and frequencies. Thus, the data available for age was binned, which enabled estimation of mean age using Sheppard’s correction [84].

## Results

### Search results

The search process is summarized in Fig. 1. For details on the excluded articles see Additional file 3.

### Characteristics of included studies

The characteristics of the included studies can be found in Table 1. The included articles were all published between 2015 and 2021 and are all written in English. From the six included studies, four included adults and two included children. All OI types (with exception of type II because of its high mortality rate during early childhood) were present. However, only one study included type V in children [96, 97] and in adults [67] and thus, no SMD could be calculated. Overall, 760 adults with OI were included in the present analyses including 481 women and 279 men. In the adult population, individuals diagnosed with type I were the most common (*N* = 483), followed by IV (*N* = 167), III (*N* = 110). The adult norm population consisted of 16,696 participants from which 8025 were women and 6893 were men.

**Table 1:** Main characteristics of the studies included

Author, year (origin)	NG author, year (origin)	Group of comparison	Sample size (n female)					Mean age in years		QoL measure
			OI total	OI I	OI III	OI IV	NG/HC	OI	NG/HC	
Gooijer et al. [32] (Netherlands)	Aaronson et al. [1] (Netherlands)	OI type I, III, IV and norm group	322 (189)	220 (139)	40 (19)	62 (31)	1742 (766)	36	48	SF-36
Hald et al. [33] (Denmark)	Bjorner et al. [12] (Denmark)	OI type I, III, IV and norm group	84 (46)	58 (33)	11 (4)	15 (9)	4048 (2141)	45	44	SF-36
Murali et al. [67] (United States)	Gandek et al. [30] (United States)	OI type I, III, IV, and norm group	302 (207)	173 (128)	49 (32)	80 (47)	′105 (926)	36	41–48**	SF-12
Orlando et al. [69] (United Kingdom)	Jenkinson et al. [44]* (United Kingdom)	OI type I, III, IV, and norm group	52 (39)	32 (24)	10 (7)	10 (8)	8801 (4′938)	44	40 **	SF-36
Song et al. [87] (China)	Control group within study	OI type I, III, IV and healthy controls	138 (50)	73 (30)	30 (11)	35 (9)	138 (50)	10	11	PedsQL
Vanz et al. [96] (Brazil)	Klatchoian et al. [46]* (Brazil)	OI type I, III, IV, and norm group	51 (22)	26	12	13	240 (124)	11	9	PedsQL

*OI* Osteogenesis imperfecta, *PedsQL* Pediatric quality of life inventory, *NG* Norm group, *HC* Healthy controls, *SF-36 and SF-12* Short form health survey questionnaire, *QoL* Quality of life

*Studies for which original authors did not originally provide a comparison group for

**Studies for which only the age range was available

In the two studies featuring children, 189 children (117 boys, 72 girls) were included. Type I was also the most diagnosed OI type in the child sample (*N* = 99), followed by IV (*N* = 48), and III (*N* = 42). The child and adolescent norm population and the healthy controls sample consisted of 318 children (144 boys, 174 girls).

The four studies featuring adults used either the Short Form Health Survey Questionnaire, 12 items (SF-12) [30] or the Short Form Health Survey Questionnaire, 36 items (SF-36) [103] as a QoL-measure. Both studies featuring children used the Pediatric quality of life inventory (PedsQL) [98].

Gooijer et al. [32] reported two comparison groups from two different papers [1, 94]. We selected Aaronson et al. [1], because the paper by von der Zee and Sanderman [94] was not in English and therefore it was not possible to extract its information. The publications by Orlando et al. [69] among adults, and [96] among children did not include population norms. We therefore included additional comparative norms [44, 46]. The paper by Song et al. [87] among children and adolescents was the only publication that included a healthy control group, instead of community norms.

### QoL in children and adolescents with OI compared to healthy controls and norms

**Fig. 2:**
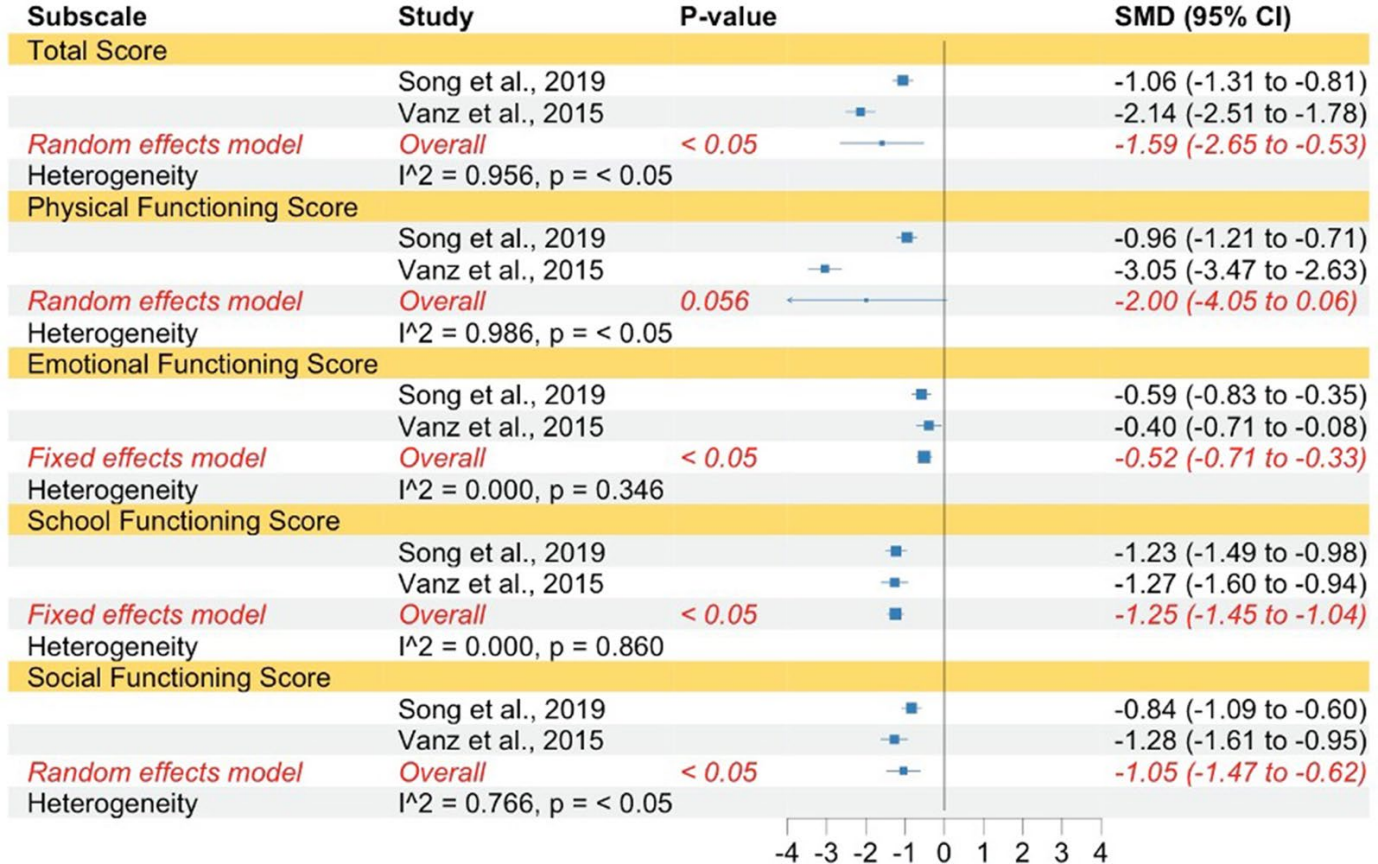
Forest plot of QoL in children with OI compared to norms. Abbreviations: *SMD* Standardized mean difference

Overall QoL levels in children and adolescents with OI were significantly lower compared to healthy controls and norms (very large effect) according to the PedsQL. The subdimension of physical functioning was not significant. However, the total score (very large), emotional functioning (medium effect), social functioning (large effect) and school functioning (very large effect) were significantly lower for children with OI. The results revealed high levels of heterogeneity for the total scale, the physical functioning subscale, and the social functioning subscale, indicating that random effect models were justified. For the emotional functioning subscale and the school functioning subscale heterogeneity was low and thus, fixed effect models were used for the analyses of the two subscales (Fig. 2).

### QoL in adults with OI compared to norms

**Fig. 3:**
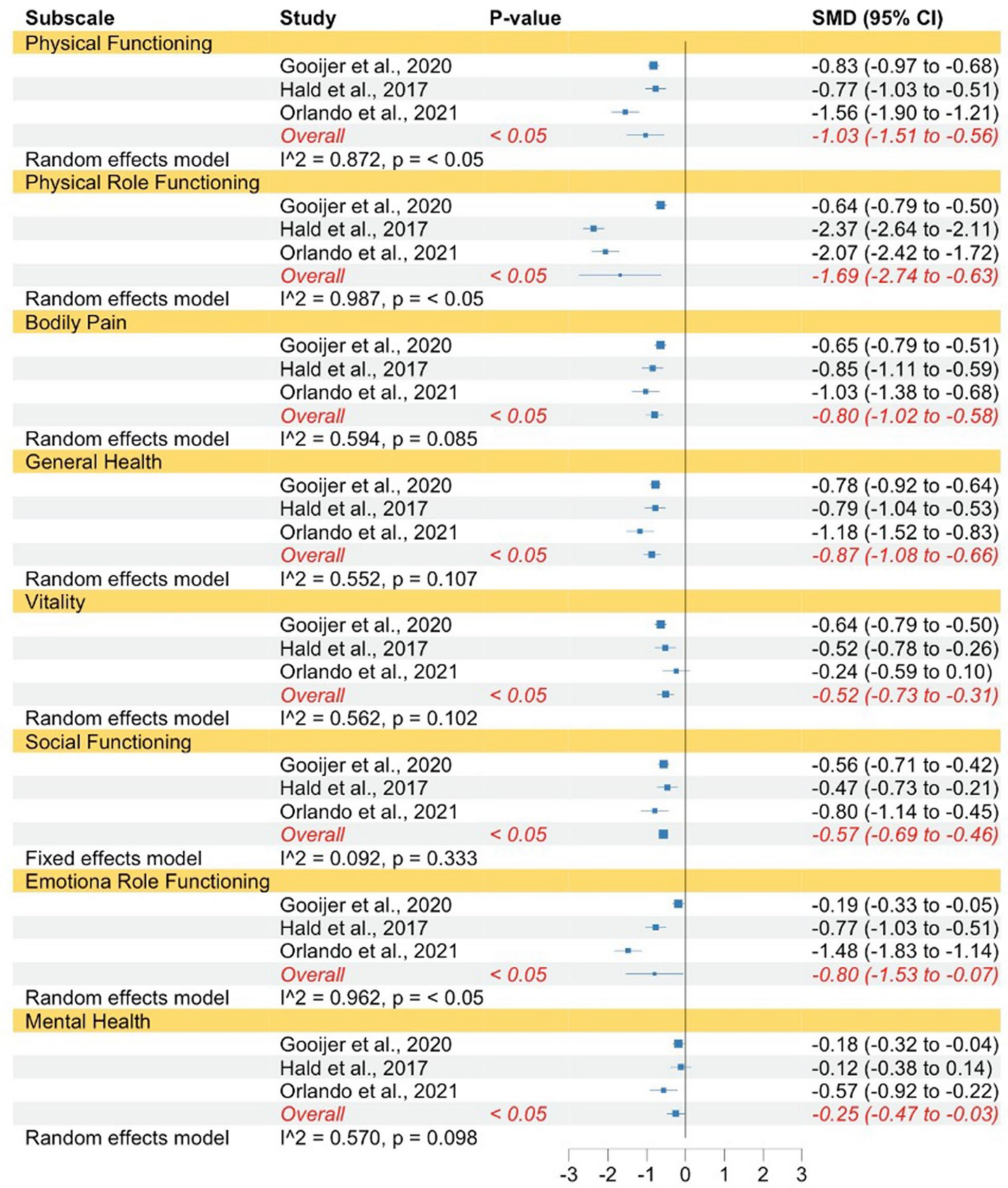
Forest plot of QoL in adults with OI type I compared to norms. Abbreviations: *SMD* Standardized mean difference

QoL in OI type I measured with the SF-12 and SF-36 was significantly lower compared to norm populations with regard to physical functioning, physical role functioning, bodily pain, general health, vitality, social functioning, emotional role functioning, and mental health (see Fig. 3). The strength of the reported effects ranged between small and very large. Almost all subscales had moderate to high levels of heterogeneity, with exception of social functioning, which justified the use of random effect models. A fixed effects model was used for social functioning.

**Fig. 4:**
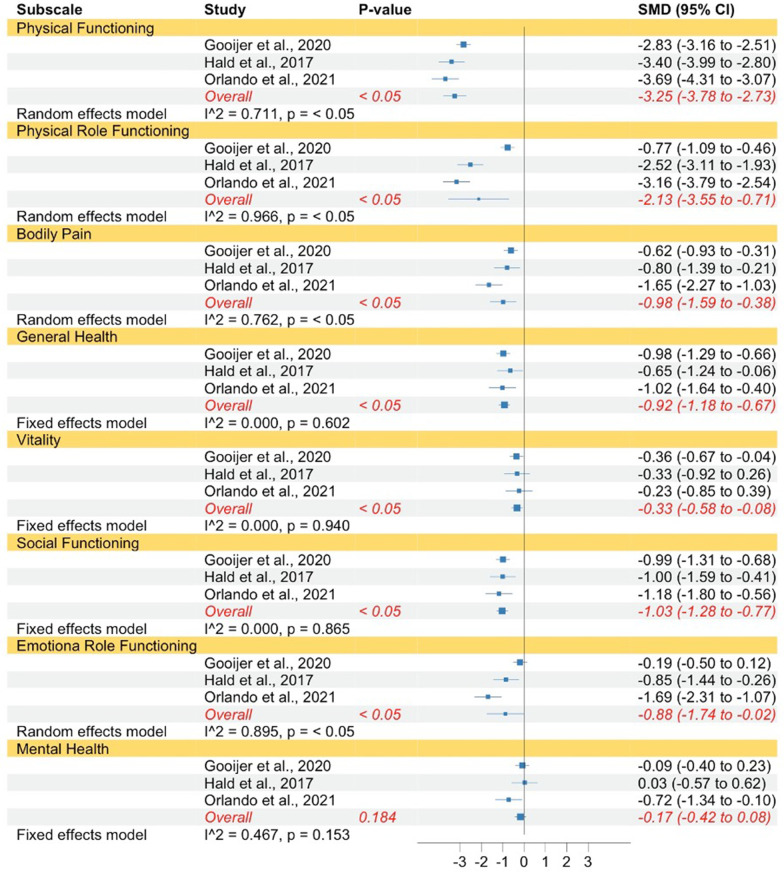
Forest plot of QoL in adults with OI type III compared to norms. Abbreviations: *SMD* Standardized mean difference

The same pattern was found when comparing individuals with OI type III to norms concerning physical functioning, physical role functioning, bodily pain, general health, vitality, social functioning, and emotional role functioning (see Fig. 4). The strength of the effects ranged between small to huge. In contrast to mental health in OI type I, individuals with OI type III did not show significantly lower mental health QoL compared to norms. The subscales physical functioning, physical role functioning, bodily pain, and emotional role functioning had high levels of heterogeneity, which justified the use of random effect models. Whereas the subscales general health, vitality, social functioning, and mental health had low levels of heterogeneity therefore, fixed-effect models were used.

**Fig. 5:**
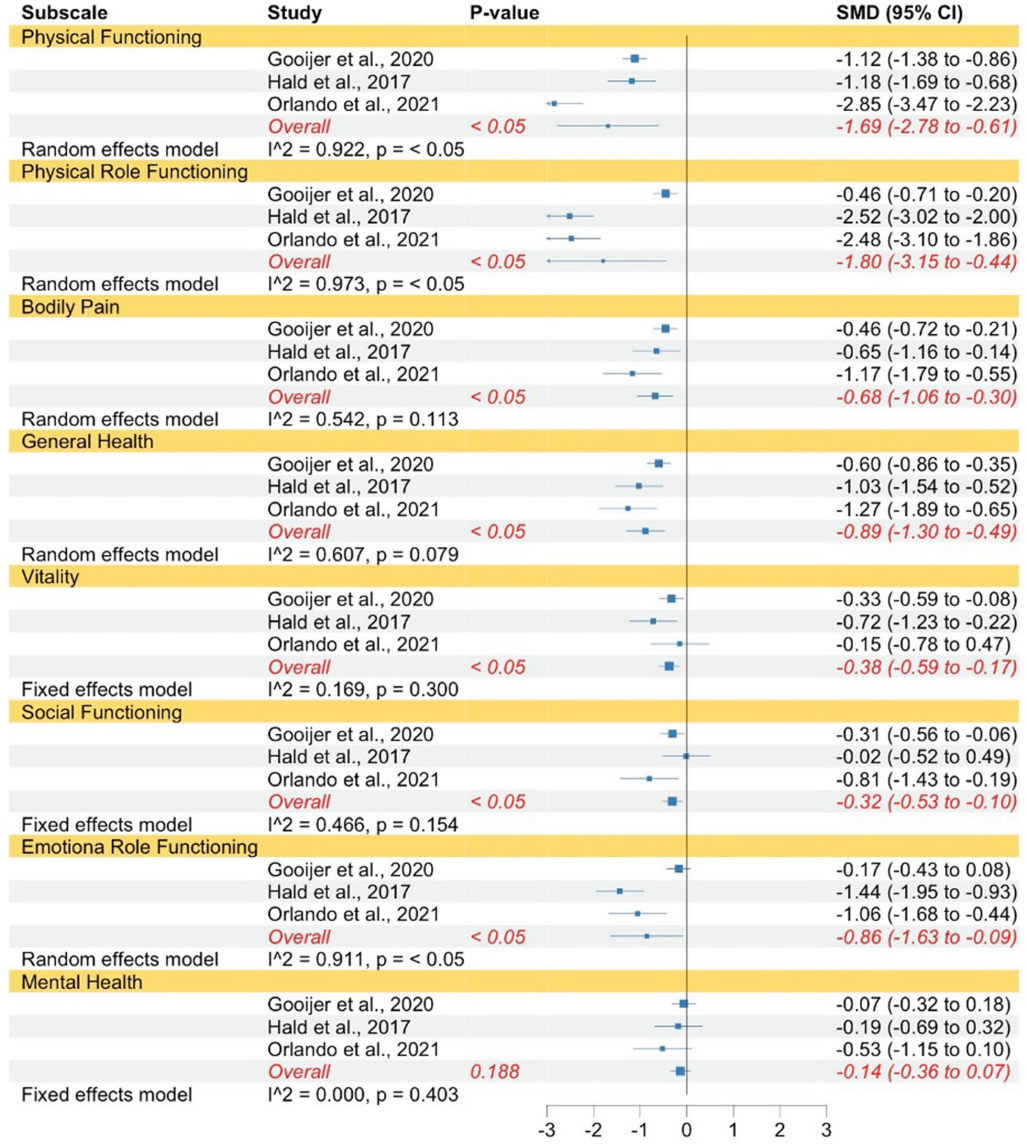
Forest plot of QoL in adults with OI type IV compared to norms. Abbreviations: *SMD* Standardized mean difference

This pattern was also found when comparing individuals with OI type IV to norms with regards to physical functioning, physical role functioning, bodily pain, general health, vitality, social functioning, and emotional role functioning (see Fig. 5). The strength of the reported effects ranged between very small and very large. In accordance with OI type III, individuals with OI type IV exhibited no significantly lower mental health levels compared to norms. All subscales had high levels of heterogeneity which justified the use of random effect models, except for vitality, social functioning, and mental health, for which fixed effect models were used.

### Comparison of QoL across OI subtypes in adults

**Fig. 6:**
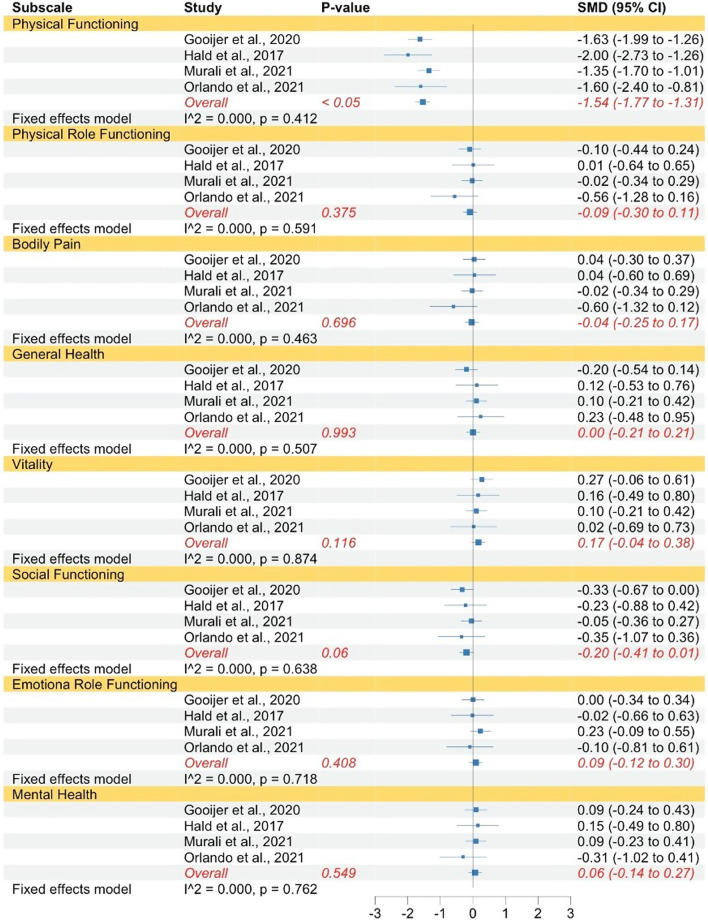
Forest plot of QoL in adults with OI type I compared to type III. Abbreviations: *SMD* Standardized mean difference

When comparing adults with OI type I to adults with type III, type III individuals had significantly lower levels of physical functioning. This was not the case for physical role functioning, bodily pain, general health, vitality, social functioning, emotional role functioning, and mental health (see Fig. 6). The strength of the reported effects ranged between very small and very large. All subscales had low levels of heterogeneity which justified the use of fixed effect models.

**Fig. 7:**
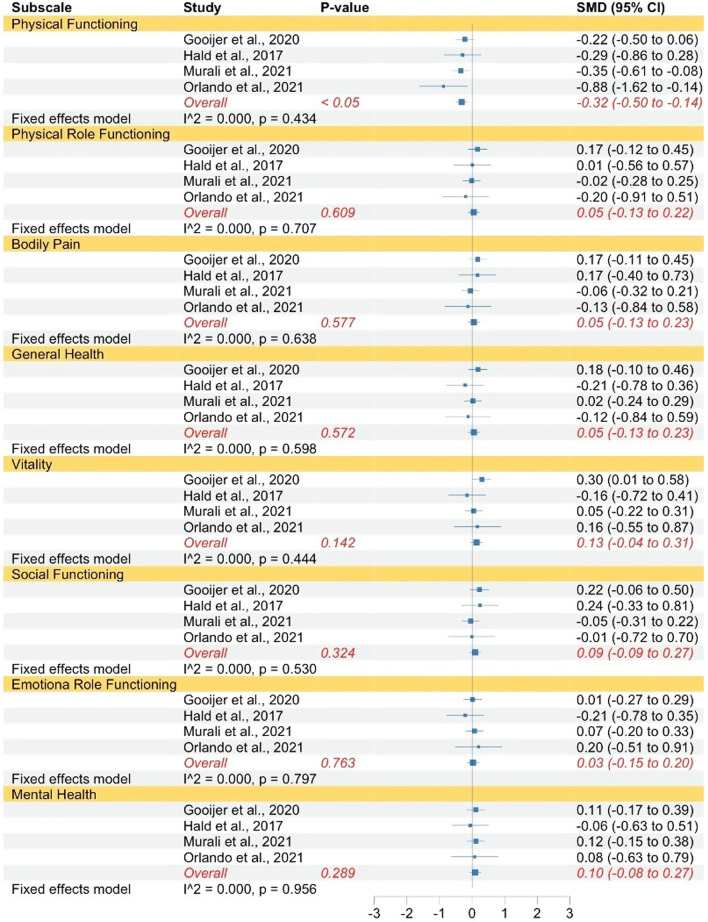
Forest plot of QoL in adults with OI type I compared to type IV. Abbreviations: *SMD *Standardized mean difference

The same pattern emerged when comparing individuals with OI type I to type IV regarding physical functioning, physical role functioning, bodily pain, general health. Vitality, emotional role functioning, and mental health (Fig. 7). The strength of the reported effects ranged between very small and large. All the subscales had low levels of heterogeneity which justified the use of fixed effect models.

When comparing adults with type III–type IV, individuals with type III had significantly lower levels in physical and social functioning. This was not the case for the SF-12 and SF-36 subscales physical role functioning, bodily pain, general health, vitality, emotional role functioning, and mental health (Additional file 4). The strength of the reported effects ranged between very small and very large. All subscales had low levels of heterogeneity which justified the use of fixed effect models.

### Meta-regression of adult OI sample

Details regarding the exploratory meta-regression are shown in Additional file 5. Results show that higher age and a higher proportion of females were significant moderators when comparing OI samples to norms. No potentially relevant variables were identified that influence the effects that OI has on QoL, when comparing subtypes among each other.

## Discussion

### QoL in children and adolescents with OI

The first objective of this meta-analysis was to investigate whether and how children and adolescents with OI differ from healthy controls and norms in terms of QoL. We found two articles comparing QoL of patients to healthy controls and norms. Children and adolescents with OI had significantly lower QoL in the domains of emotional, school, and social functioning. This was also mirrored by a significantly lower total score with a very large effect size. The effect for emotional functioning was of medium strength, whereas the effect for school functioning was very large, and the effect for social functioning was large. However, due to limited data availability, the difference between different OI types could not be examined in detail. As the analyses conducted for the adult sample show, the results can look very different when the types of OI are considered separately. As shown previously in single studies, differences between OI types are also evident in childhood and adolescence [87, 96, 97]. Children with type III and IV and V exhibited lower levels of physical and school functioning, while emotional and social functioning was lowest in type III, followed by type I. Emotional functioning was lowest in type I, followed by type III. Thus, it is possible that the lower levels regarding school functioning are driven by type III, IV, and V, whereas lower levels of emotional functioning are influenced by the type I and III. Consequently, the present results must be considered with caution, as the direction and strength of the effect could be different for each OI type.

Another important aspect is that the two studies included in the analysis contained both children and adolescents [87, 96, 97]. However, these two groups face different challenges and have different needs. Adolescents face various obstacles during and after puberty as they are confronted with physical and social transitions [89]. Due to these challenges, adolescents generally have lower levels of QoL compared to children as reported from epidemiological studies [62]. In addition, sex differences become more important with age due to biological, psychological and social changes [60, 71]. Previous studies suggest that female adolescents are more prone to mental health disorders, complaints about their mental health and poorer perceptions of their general health compared to male adolescents [19, 29]. For these two reasons, the observed effects might be different if the two genders were considered separately.

Another aspect to consider is that one of the included studies used self-report versions of the PedsQL [96, 97], while the other study included both proxy and self-report [87]. There is increasing evidence that patients are experts on their own health [11, 17]. Patient reports from adults are already a vital part of their symptom assessment [6]. However, this is more challenging in children, as only few valid assessment tools are available [3, 106]. As a result, proxy reports from caregivers are used to either fully, or partially replace children’s self-reports. Previous findings suggest that proxy reports deviate from children’s perceptions [20, 48, 53, 98]. Agreement between parent and child is influenced by several variables, among them the domain being measured [24]. Physical aspects are rated more similar by both parties, whereas emotional and social aspects, are rated lower by parents. This is particularly important for chronically ill children, because parents tend to rate their child’s QoL too low [15, 80]. Therefore, the present results might be different if child self-reports and parental proxy-reports were considered separately. However, due to the limited number of studies in this age group this was not possible.

In summary, the results suggest that children and adolescents with OI perform significantly worse in emotional, school functioning and social functioning compared to healthy controls and norms, which is also mirrored by the total score. This has also been found in other rare pediatric connective tissue disorders such as Marfan syndrome, Ehlers–Danlos syndrome, and skeletal dysplasia [34]. School is an important part of a child's daily life and provides ample opportunities for learning new content, as well as for social learning and emotional development [4]. Based on the present findings, it appears that past efforts have focused on improving physical functioning rather than the emotional, school, and social functioning of children with OI [45]. This is also due to the burden of treatment schedules with numerous medical appointments that can conflict with regular school attendance. It is of great importance to help parents and children find an optimal balance between the different areas of a child's daily life.

### QoL in adults with OI

The second goal of this meta-analysis was to examine whether and how adults with OI differ from healthy norms in terms of QoL and if there are differences across OI subtypes. Regardless of the type of OI, adults performed significantly worse on all physical component subscales compared to norms, with effect sizes ranging between large and huge. When comparing subtypes, type IV with a small effect size and type III with a very large effect size, had significantly lower levels of physical functioning than type I. Individuals with type III had significantly lower physical functioning levels compared to individuals with type IV, with a very large effect size which is most probably a result of the physical impairments and problems associated with the more severe types of OI. These findings are comparable to other chronic and rare connective tissue diseases such as Ehlers–Danlos syndrome and Marfan syndrome [9, 75].

Regarding the mental health dimension of QoL, individuals with type I, were the only ones who scored significantly lower compared to norms. However, this effect was small. In contrast, adults with type III and IV, did not have significantly worse mental health scores, although comparison across subtypes revealed that the two types had significantly worse physical functioning levels. These results suggest that the severity of the disease itself and its accompanying physical impairments need not automatically lead to poorer mental health. Conversely, a milder manifestation of the disease does not lead to better mental health. At first glance, this finding is counterintuitive, as adults with type III and IV face severe physical limitations and deformities, and individuals with type III also have a higher early mortality rate [26]. Good mental QoL in the face of severe physical impairments is a well-known phenomenon called the disability paradox, which has also been found in other populations with chronic diseases [2]. There are several possible explanations for this phenomenon, the first one being the so-called response shift. Response shift is defined as the change in internal concepts, standards, and values of an individuals’ QoL perceptions [85]. By adjusting standards over time, i.e. making a response shift, a person with physical impairments can maintain his or her level of QoL whilst living with an illness. Thus, response shifts help patients adapt to changing health conditions without compromising their QoL or well-being [88]. Interestingly, response shift has not been studied in rare diseases. A previous longitudinal study by Seery et al. [86], however, shows that response shift might be an important factor in individuals with OI. As mentioned earlier, an important difference between the type I and the other types, is the timepoint of diagnosis. Individuals with type III, IV and V tend to be diagnosed much earlier and therefore, have to cope with their disease earlier. Seery et al. [86] showed that people who have experienced adversity report better mental health and well-being at follow-up compared to individuals with no adverse experiences. This particular finding illustrates that a certain amount of lifetime adversity can actually be beneficial and even lead to someone being more equipped to cope with future adverse events. Research concerning response shift may provide a possible explanation for why different degrees of physical impairment do not affect all aspects of QoL equally. Because individuals with type I often do not receive their diagnosis until adulthood, they may not have as much time to adjust as participants with type III, IV, and V who are typically diagnosed in infancy or early childhood.

A second potential explanation for why individuals with type I exhibit worse mental QoL compared to type III, IV, and V could be because of symptom invisibility. People with type III and IV face visible deformations, such as scoliosis, dwarfism, or malformations of the skull. Therefore, physical symptoms are more easily perceived from the outside and patients may receive more support and less social devaluation from those around them. Previous research on chronic pain has found that invisibility of symptoms can lead to social stigmatization and thus lower mental health and self-esteem [102]. For another sample of rare disease individuals, namely Ehlers-Danlos patients, those who did not have observable symptoms, often felt left behind by their health-care providers and thus, struggled to find a physician with whom they are able to talk about their symptoms openly [8]. The invisibility of the disease itself can lead to stigma expressed by the medical team [10], the educational environment [51] and by peers [102]. Such experiences related to stigma can negatively impact an individuals’ health [7], QoL [56], and mental health [56]. Due to the barely visible physical symptoms of type I, it can be considered as an invisible disease. Future research should investigate whether lower mental health scores are due to stigmatization by the patient's environment, such as peers or medical providers.

According to the minority stress model, the concept of disability is determined not only by medical perceptions but also by society's restrictive perceptions of what is considered normal [77]. The model in question states that minorities are exposed to a unique set of stressors related to their minority identity that results from a conflict between the prevailing values of the minority and the values of society at large [61]. These stressors may subsequently have a negative impact on the health of the minority in question through distal stressors such as discrimination and prejudice or proximal stressors, i.e., internalized stereotypes. To avoid such stressors, minorities often try to hide the characteristics that distinguish them from the normal population, which can lead to deteriorating mental and physical health. In fact, studies show that people with rare genetic conditions often avoid telling others about their diagnosis because they want to protect themselves from social stigma [100]. In general, stigma and social misperceptions are common problems in the rare disease community [101]. People with rare diseases believe that these public misperceptions are due to societal attitudes and the general population’s lack of knowledge about rare diseases, leading to feelings of discrimination and social exclusion. In accordance with the minority stress model, people with OI describe having a strong desire to be perceived and treated as normal [91]. It could be argued that in the case of OI Type I, differences in normality are more easily concealed, causing even more stress to this subtype because of having to pretend to fit into the normality category of a particular society even though they do not actually belong, leading to increased stress. The various processes described in this model are a promising potential explanation for the relevant contribution of societal perceptions to the poorer mental health of OI Type I.

Depending on the type of OI, the impact of the disease on mental health may vary. Meta-regression analyses showed that the proportion of females had a decreasing impact on physical functioning, physical role functioning general health and emotional role functioning. However, the results regarding the meta-regression should be considered with caution, as the sample size of women within the different OI types was rather small. According to the results of the meta-regressions performed, higher age seemed to contribute to a decrease in physical role functioning, bodily pain, general health, and emotional role functioning. People living with a rare disease typically face challenges with the diagnostic process, the paucity of treatment options, and the small number of specialists. These challenges are even more pronounced the older a person with a rare disease such as OI becomes. Therefore, it comes as no surprise that older age seems to contribute negatively towards several aspects of QoL [5, 81].

### Strengths and limitations

A quantitative comparison between a patient population and norms, as well as healthy controls, is of great importance to understand in which areas care and treatment might be optimized. A meta-analysis produces a clear effect size estimate and can lead to a conclusive summary across different inconclusive single studies [66]. In addition, meta-analyses and systematic reviews contain a more extensive range of patients compared to singles studies [65]. Thus, a meta-analysis can better support clinical and scientific decision making than individual studies, as it is associated with greater confidence when applying such results to patients. We also searched a total of nine databases, including all databases recommended for investigating clinical research questions [36, 40, 66]. Additionally, two of the databases searched were trial registries (Cochrane Central Register of Controlled Trials and Clinicaltrials.gov). Furthermore, the reference lists of included studies were searched, and unpublished data were requested from various experts in the field to obtain the largest number of studies possible. Two reviewers were involved in study selection and extraction, increasing the confidence and reproducibility of the process.

The main limitation of this meta-analysis was that the population of interest is a rare disease and thus only a limited number of studies and participants was available [28]. In addition, smaller samples, which are typical in rare diseases, lead to a greater heterogeneity across studies [41]. The combination of a small number of studies and small sample sizes therefore leads to difficulties in the estimation of between-study heterogeneity and undermines the possibility to estimate publication bias. In the case of the present meta-analysis, study designs often differed, for example, in terms of different types of control groups (healthy controls vs. norm populations) or sources of reporting (self-report vs. proxy report), which increased between-study heterogeneity. In addition, it is relevant to point out that OI is a collective term for a very heterogeneous group of connective tissue syndromes and that its classification has evolved in recent years [22, 95]. New genetic discoveries led to a new classification that addresses both clinical and genetic scientific findings. These newly added OI types resulted in a classification system where types are not mutually exclusive. This also resulted in people inaccurately typed and sometimes not even assigned to a specific subtype. Accordingly, there is a possibility that the individuals included in the present meta-analysis were assigned to an incorrect subtype and thus, leading to a higher between-study heterogeneity because the categorization might have been done differently. We took this factor into account by estimating the CIs of SMDs and by evaluating risk of bias, which makes the confidence concerning the estimates of effects accessible and more credible [66]. Additionally, subgroup analyses and meta-regressions were performed in order to explain heterogeneity.

Another limitation of the present study is that we were not able to examine clinically meaningful differences in QoL. A small difference between groups may reach statistical significance. However, a small effect is unlikely to be clinically important, or from the perspective of the clinician, a difference that makes a particular treatment worthwhile [43]. A solution is the use of the MCID, which refers to the smallest difference of a score that is considered to be of importance to the patient [70]. Unfortunately, in the OI population, there is no data on MCIDs for the SF-36, SF-12 and the PedsQL. Identifying an MCID in the context of OI in QoL would facilitate interpretation of effects and thus enhance the understanding by researchers and clinicians. A previous study showed that individuals with OI had significantly lower scores in anxiety and general mental health, but that these scores did not reach clinical relevance [90]. Accordingly, the results of the present analysis might have been different if viewed through the lens of clinical relevance. Nevertheless, we used the standard rules of thumbs for SMDs [83] in order to aid interpretation of our results.

A limitation and at the same time a strength of the present work are the norm comparisons. Norms are a pertinent reference point because they allow comparison between individuals with a disease and the general population, which helps to make inequalities visible, making norms essential for identifying groups with lower quality of life and thus capturing disease burden [57, 74]. However, if a disease group is to be compared with norms, a disease-specific measure cannot be used, preventing the measurement of disease-specific attributes that are relevant to the population of people with chronic conditions [49].

### Future research

The present meta-analysis shows that a variety of dimensions of QoL can be impaired in individuals with OI, regardless of their age. OI seems to negatively impact the lives’ of patients similar to other chronic diseases [34].

The inclusion of a wider array of patient-reported outcome measures (PROMs) could shed a light upon the underlying mechanisms between OI and QoL such as individual risk and protective factors. One risk factor that is often overlooked in OI research is pain. Many people with OI suffer from acute and chronic pain as a result of fractures and scoliosis [59]. Chronic pain causes additional stress in a population already struggling with severe limitations [13]. Another important factor is the higher prevalence rates of anxiety and affective disorders in the group of people with rare diseases [92]. The combination of a mental and a somatic disorder can lead to decreased QoL and a worse prognosis [21]. A better understanding of these risk and protective factor is critical to improving the overall health and QoL of people with OI. Also, it would be of interest to use different QoL measures because different authors have defined QoL in various ways, with some focusing more on functional status and others focusing more on subjective well-being, which is subsequently reflected in QoL scales [64]. The present meta-analysis showed a significant difference in mental health on OI Type I and norms, however, both the SF-36 and SF-12 were not specifically designed for assessing mental health. In fact, previous studies have shown that the mental health subscale of the SF-36 and the SF-12 is overly sensitive and exhibits ceiling effects. Future studies should attempt to replicate the finding in questions and combine measures developed for mental health assessment with QoL measures. In addition, future research should focus on psychometric validation of PROMS in the OI population, without which psychometric performance cannot be guaranteed.

Variables related to care- and treatment-related factors, such as treatment burden, might also have explanatory value because people with OI are highly burdened with treatment [82]. More people with a rare disease report financial burdens because not all of their expenses are covered by health insurance [92]. Patients with rare diseases also report that they lack information about their disease. Results show that low levels of health literacy are associated with lower quality of life and self-efficacy [52]. Therefore, factors related to care and treatment are a promising path toward understanding the relationship between OI and quality of life.

However, not only PROMs at the patient level, but also so-called observer-reported outcome measures (ObsPROMs) are of importance. Higher levels of pain and impaired physical function in children with OI have been associated with higher stress, quality of life, and depression in parents [47]. Children with OI have been found to downplay their pain to protect their parents in order to shield them from the impacts of their illness [91]. These findings indicate the need to look more closely at caregiver needs, as they have high explanatory value for the association between OI in children and lower QoL.

In addition to studying the factors that might contribute to lower QoL in different types of OI, it is of great importance to also study the evolution of QoL in terms of developmental changes and long-term effects. This would also allow the examination of the relationship between explanatory variables such as diagnostic uncertainty, response shift, and invisibility of symptoms. In addition, longitudinal data could help clarify the impact of parental perceptions, as proxy reports often differ from children’s self-reports.

As mentioned beforehand conducting research in rare diseases is challenging due to small sample sizes, a possible solution to this barrier are multicentric and international studies and collaboration with patient organizations. Especially data on children and adolescents is scarce, but highly relevant due to the early onset of OI. Future studies should attempt to collaborate with other centers and OI patient organizations to achieve larger sample sizes. Another possible solution to this problem is the use of Open Science, as this promotes the exchange of data [79].

### Clinical implications

The results of the present meta-analysis show a clear pattern. Since mental health appears to be compromised in individuals with OI, we suggest that all patients should be routinely screened for mental health regardless of subtype. In addition, children should be supported in their respective schools because they have shown poorer functioning in school. Previous findings on school support have shown that such intervention also promote emotional and social functioning, which is due to the interaction of the three constructs [105]. Early screening and intervention may prevent at-risk individuals from developing a chronic, lifelong mental disorder. This is especially important for children, as it is more difficult for them to ask for help even when lower mental health impairs their daily functioning. Because social functioning has been shown to be impaired in OI type I, III and IV, social interventions might be especially helpful. Physical activity interventions have also shown promising results in terms of physical and mental QoL [93]. We consider it to be important to provide sufficient support to patients and their families in a comprehensive way, complementing medical interventions by also adding psychological and social interventions [76].

## Conclusion

The present meta-analysis allowed for deeper insight into the QoL of pediatric and adult OI patients. Our findings underline the importance of paying attention to potential QoL impairments in OI. The results show that the clinical severity of an OI diagnosis is not associated with impairments in mental health. Therefore, it is important that future research considers different OI types and QoL dimensions separately. Children with OI reported lower levels of QoL regarding emotional, school, and social functioning when compared to healthy controls and norm groups. Adults across all OI types reported significantly worse physical QoL components. Only adults with OI type I reported significantly worse QoL across all mental components. Further research is needed to explain the lack of the relation between clinical severity and QoL. In addition, future research should explore potential factors that influence QoL such as diagnostic uncertainty and coping mechanisms.

## Supplementary Information

**Additional file 1**. Checklist to assess study quality, adapted critical appraisal checklist for analytical cross-sectional studies.

**Additional file 2**. Risk of bias summary barplot.

**Additional file 3**. Reference list of excluded articles and justification for exclusion.

**Additional file 4**. Forest plot of QoL in adults with OI type III compared to type IV.

**Additional file 5**. Meta-regression of moderators of effects of QoL of adults with OI compared to norms.

## Data Availability

The dataset supporting the conclusions of this article is available in the Open Science Framework repository, https://osf.io/a4dxb/?view_only=efbaa104c7c84a55b639c665059912e1.

## Abbreviations

CI: Confidence interval
MCID: Minimal clinically important difference
OI: Osteogenesis imperfecta
ObsPROMs: Observer-reported outcome measures
PedsQL: Pediatric quality of life inventory
PROMs: Patient-reported outcomes
REML: Restricted maximum likelihood estimator
QoL: Quality of life
SMD: Standardized mean difference
SF: Short form Health Survey Questionnaire
