# Supplementary material for: Quality of life of pediatric and adult individuals with osteogenesis imperfecta: a meta-analysis
Source: Orphanet J Rare Dis. 2023 May 24;18:123. doi: 10.1186/s13023-023-02728-z (PMC10207627; doi:10.1186/s13023-023-02728-z)
Supplement: Supplementary file 1 — Additional file 1. Checklist to assess study quality, adapted critical appraisal checklist for analytical cross-sectional studies. [file 13023_2023_2728_MOESM1_ESM.docx]

**Additional File 1.** Checklist to assess study quality, adapted critical appraisal checklist for analytical cross-sectional studies

Reviewer:

Author:

|  | Risk of Bias | | | |
| --- | --- | --- | --- | --- |
|  | High | Some concern | Low | No information |
| 1. Were the criteria for inclusion and exclusion in the sample clearly defined? | □ | □ | □ | □ |
| 1. Were the groups comparable other than the presence of disease in cases or the absence of disease in controls / subtypes? | □ | □ | □ | □ |
| 1. Were the study subjects and the setting described in detail? | □ | □ | □ | □ |
| 1. Was quality of life measured in a valid and reliable way? | □ | □ | □ | □ |
| 1. Was quality of life measured in the same way for cases and controls / subtypes? | □ | □ | □ | □ |
| 1. Was appropriate statistical analysis used? | □ | □ | □ | □ |
