# Supplementary figures and images for: Quality of life of pediatric and adult individuals with osteogenesis imperfecta: a meta-analysis
Source: Orphanet J Rare Dis. 2023 May 24;18:123. doi: 10.1186/s13023-023-02728-z (PMC10207627; doi:10.1186/s13023-023-02728-z)

**Additional File 2.** Risk of bias summary barplot

**
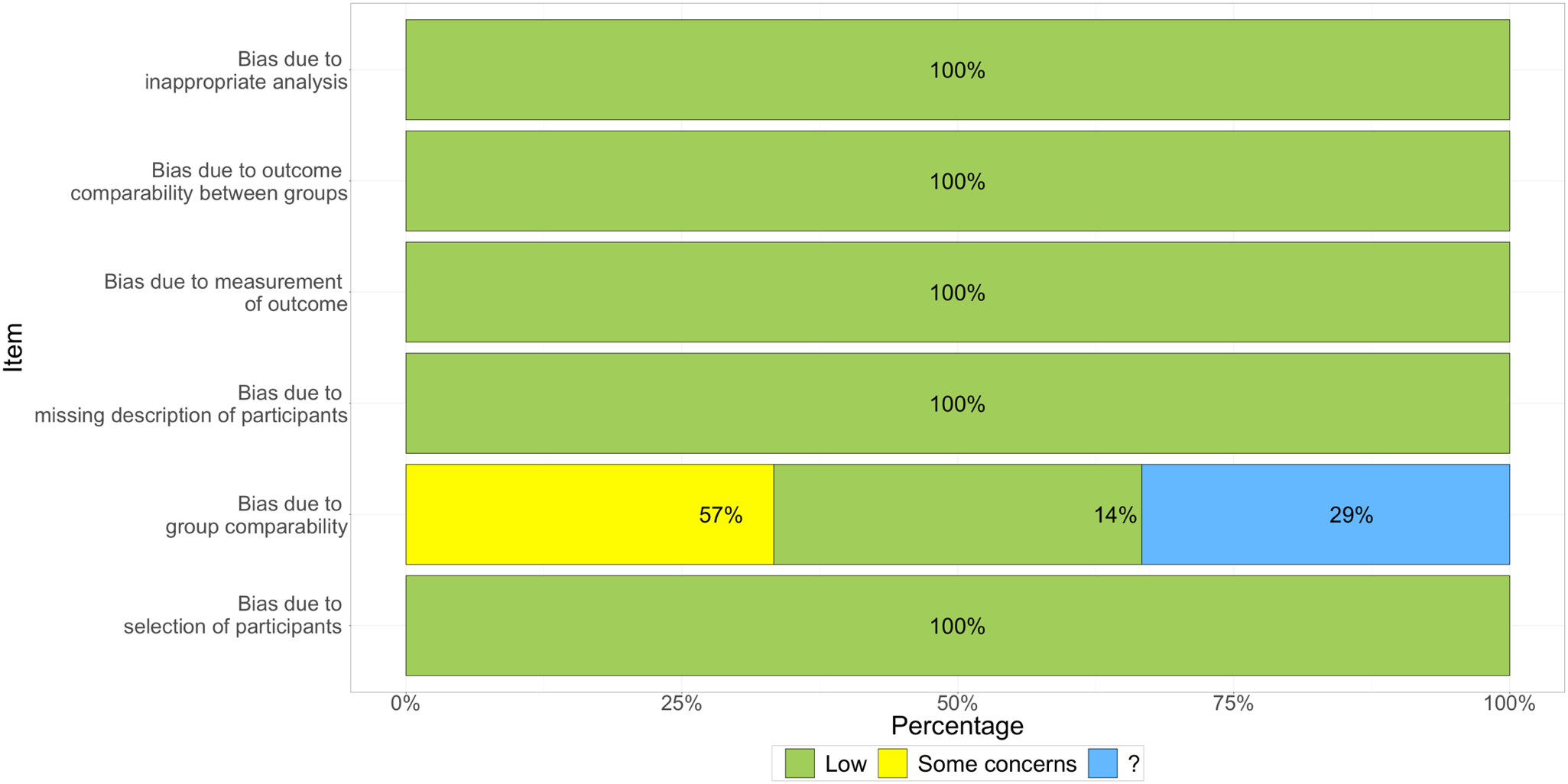
**

Supplement: Supplementary file 2 — Additional file 2. Risk of bias summary barplot. [file 13023_2023_2728_MOESM2_ESM.docx]
