# Supplementary material for: Quality of life of pediatric and adult individuals with osteogenesis imperfecta: a meta-analysis
Source: Orphanet J Rare Dis. 2023 May 24;18:123. doi: 10.1186/s13023-023-02728-z (PMC10207627; doi:10.1186/s13023-023-02728-z)
Supplement: Supplementary file 3 — Additional file 3. Reference list of excluded articles and justification for exclusion. [file 13023_2023_2728_MOESM3_ESM.docx]

**Additional File 3.** Reference list of excluded articles and justification for exclusion

**No standardized or generic quality of life measure (n = 9)**

Ashby, E., Montpetit, K., Hamdy, R. C., & Fassier, F. (2018). Functional outcome of humeral rodding in children with osteogenesis imperfecta. *Journal of Pediatric Orthopaedics*, *38*(1), 49-53. <https://doi.org/10.1097/BPO.0000000000000729>

Ashby, E., Montpetit, K., Hamdy, R. C., & Fassier, F. (2018). Functional outcome of forearm rodding in children with osteogenesis imperfecta. *Journal of Pediatric Orthopaedics*, *38*(1), 54-59. <https://doi.org/10.1097/BPO.0000000000000724>

Caudill, A., Flanagan, A., Hassani, S., Graf, A., Bajorunaite, R., Harris, G., & Smith, P. (2010). Ankle strength and functional limitations in children and adolescents with type I osteogenesis imperfecta. *Pediatric Physical Therapy*, *22*(3), 288-295. https://doi.org/10.1097/PEP.0b013e3181ea8b8d

Graf, A., Hassani, S., Krzak, J., Caudill, A., Flanagan, A., Bajorunaite, R., ... & Smith, P. (2009). Gait characteristics and functional assessment of children with type I osteogenesis imperfecta. *Journal of Orthopaedic Research*, *27*(9), 1182-1190. <https://doi.org/10.1002/jor.20871>

Huang, R. P., Ambrose, C. G., Sullivan, E., & Haynes, R. J. (2006). Functional significance of bone density measurements in children with osteogenesis imperfecta. *The Journal of Bone and Joint Surgery. American Volume*, *88*(6), 1324–1330. https://doi.org/10.2106/JBJS.E.00333

Murali, C. N., Cuthbertson, D., Slater, B., Nguyen, D., Turner, A., Harris, G., Sutton, V. R., Lee, B., Members of the BBD Consortium, & Nagamani, S. (2020). Pediatric Outcomes Data Collection Instrument is a Useful Patient-Reported Outcome Measure for Physical Function in Children with Osteogenesis Imperfecta. *Genetics in Medicine: Official Journal of the American College of Medical Genetics*, *22*(3), 581–589. https://doi.org/10.1038/s41436-019-0688-6

Seikaly, M. G., Kopanati, S., Salhab, N., Waber, P., Patterson, D., Browne, R., & Herring, J. A. (2005). Impact of alendronate on quality of life in children with osteogenesis imperfecta. *Journal of Pediatric Orthopedics*, *25*(6), 786–791. https://doi.org/10.1097/01.bpo.0000176162.78980.ed

Sousa, T., Bompadre, V., & White, K. K. (2014). Musculoskeletal functional outcomes in children with osteogenesis imperfecta: associations with disease severity and pamidronate therapy. *Journal of Pediatric Orthopedics*, *34*(1), 118–122. https://doi.org/10.1097/BPO.0b013e3182a006a0

Yonko, E. A., Emanuel, J. S., Carter, E. M., Sandhaus, R. A., & Raggio, C. L. (2020). Respiratory impairment impacts QOL in osteogenesis imperfecta independent of skeletal abnormalities. *Archives of Osteoporosis*, *15*(1), 153. https://doi.org/10.1007/s11657-020-00818-0

**No report of mean and / or standard deviation (n = 11)**

Aubry-Rozier, B., Richard, C., Unger, S., Hans, D., Campos-Xavier, B., Schneider, P., Paquier, C., Pasche, J., Bonafé, L., & Bregou, A. (2020). Osteogenesis imperfecta: towards an individualised interdisciplinary care strategy to improve physical activity and quality of life. *Swiss Medical Weekly*, *150*, w20285. <https://doi.org/10.4414/smw.2020.20285>

Balkefors, V., Mattsson, E., Pernow, Y., & Sääf, M. (2013). Functioning and quality of life in adults with mild-to-moderate osteogenesis imperfecta. *Physiotherapy Research International: The Journal for Researchers and Clinicians in Physical Therapy*, *18*(4), 203–211. https://doi.org/10.1002/pri.1546

Engelbert, R. H. H., Custers, J. W. H., Van Der Net, J., Van Der Graaf, Y., Beemer, F. A., & Helders, P. J. M. (1997). Functional outcome in osteogenesis imperfecta: disability profiles using the PEDI. *Pediatric Physical Therapy*, *9*(1), 18-22.

Engelbert, R. H., Gulmans, V. A., Uiterwaal, C. S., & Helders, P. J. (2001). Osteogenesis imperfecta in childhood: perceived competence in relation to impairment and disability. *Archives of Physical Medicine and Rehabilitation*, *82*(7), 943–948. https://doi.org/10.1053/apmr.2001.23889

Engelbert, R. H., Uiterwaal, C. S., Gerver, W. J., van der Net, J. J., Pruijs, H. E., & Helders, P. J. (2004). Osteogenesis imperfecta in childhood: impairment and disability. A prospective study with 4-year follow-up. *Archives of Physical Medicine and Rehabilitation*, *85*(5), 772–778. https://doi.org/10.1016/j.apmr.2003.08.085

Nicolaou, N., Bowe, J. D., Wilkinson, J. M., Fernandes, J. A., & Bell, M. J. (2011). Use of the Sheffield telescopic intramedullary rod system for the management of osteogenesis imperfecta: clinical outcomes at an average follow-up of nineteen years. *The Journal of Bone and Joint Surgery. American Volume*, *93*(21), 1994–2000. https://doi.org/10.2106/JBJS.J.01893

Tosi, L. L., Oetgen, M. E., Floor, M. K., Huber, M. B., Kennelly, A. M., McCarter, R. J., Rak, M. F., Simmonds, B. J., Simpson, M. D., Tucker, C. A., & McKiernan, F. E. (2015). Initial report of the osteogenesis imperfecta adult natural history initiative. *Orphanet Journal of Rare Diseases*, *10*, 146. <https://doi.org/10.1186/s13023-015-0362-2>

Tosi, L. L., Floor, M. K., Dollar, C. M., Gillies, A. P., Members of the Brittle Bone Disease Consortium, Hart, T. S., Cuthbertson, D. D., Sutton, V. R., & Krischer, J. P. (2019). Assessing disease experience across the life span for individuals with osteogenesis imperfecta: challenges and opportunities for patient-reported outcomes (PROs) measurement: a pilot study. *Orphanet Journal of Rare Diseases*, *14*(1), 23. https://doi.org/10.1186/s13023-019-1004-x

Van Brussel, M., Takken, T., Uiterwaal, C. S., Pruijs, H. J., Van der Net, J., Helders, P. J., & Engelbert, R. H. (2008). Physical training in children with osteogenesis imperfecta. *The Journal of Pediatrics*, *152*(1), 111-116.

Widmann, R. F., Bitan, F. D., Laplaza, F. J., Burke, S. W., DiMaio, M. F., & Schneider, R. (1999). Spinal deformity, pulmonary compromise, and quality of life in osteogenesis imperfecta. *Spine*, *24*(16), 1673–1678. <https://doi.org/10.1097/00007632-199908150-00008>

Widmann, R. F., Laplaza, F. J., Bitan, F. D., Brooks, C. E., & Root, L. (2002). Quality of life in osteogenesis imperfecta. *International Orthopaedics*, *26*(1), 3–6. https://doi.org/10.1007/s002640100292

**Not enough data on scale available (less than two studies used the scale in question) (N = 2)**

Forestier-Zhang, L., Watts, L., Turner, A., Teare, H., Kaye, J., Barrett, J., Cooper, C., Eastell, R., Wordsworth, P., Javaid, M. K., & Pinedo-Villanueva, R. (2016). Health-related quality of life and a cost-utility simulation of adults in the UK with osteogenesis imperfecta, X-linked hypophosphatemia and fibrous dysplasia. *Orphanet Journal of Rare Diseases*, *11*(1), 160. https://doi.org/10.1186/s13023-016-0538-4

Wiggins, S., Kreikemeier, R., & Struwe, L. (2020). Parents' Perceptions of Health-Related Quality of Life of Children Diagnosed with Osteogenesis Imperfecta. *Journal of Pediatric Nursing*, *55*, 75–82. https://doi.org/10.1016/j.pedn.2020.06.009

**No comparison group available (N = 2)**

Garganta, M. D., Jaser, S. S., Lazow, M. A., Schoenecker, J. G., Cobry, E., Hays, S. R., & Simmons, J. H. (2018). Cyclic bisphosphonate therapy reduces pain and improves physical functioning in children with osteogenesis imperfecta. *BMC Musculoskeletal Disorders*, *19*(1), 344. https://doi.org/10.1186/s12891-018-2252-y

Tsimicalis, A., Boitor, M., Ferland, C. E., Rauch, F., Le May, S., Carrier, J. I., Ngheim, T., & Bilodeau, C. (2018). Pain and quality of life of children and adolescents with osteogenesis imperfecta over a bisphosphonate treatment cycle. *European Journal of Pediatrics*, *177*(6), 891–902. https://doi.org/10.1007/s00431-018-3127-9

**OI group not listed separately (N = 1)**

Matsushita, M., Mishima, K., Yamashita, S., Haga, N., Fujiwara, S., Ozono, K., Kubota, T., Kitaoka, T., Ishiguro, N., & Kitoh, H. (2020a). Impact of fracture characteristics and disease-specific complications on health-related quality of life in osteogenesis imperfecta. *Journal of Bone and Mineral Metabolism*, *38*(1), 109–116. https://doi.org/10.1007/s00774-019-01033-9
