# Supplementary material for: Quality of life of pediatric and adult individuals with osteogenesis imperfecta: a meta-analysis
Source: Orphanet J Rare Dis. 2023 May 24;18:123. doi: 10.1186/s13023-023-02728-z (PMC10207627; doi:10.1186/s13023-023-02728-z)
Supplement: Supplementary file 4 — Additional file 4. Forest plot of QoL in adults with OI type III compared to type IV. [file 13023_2023_2728_MOESM4_ESM.docx]

**Additional File 4.** Forest plot of QoL in adults with OI type III compared to type IV


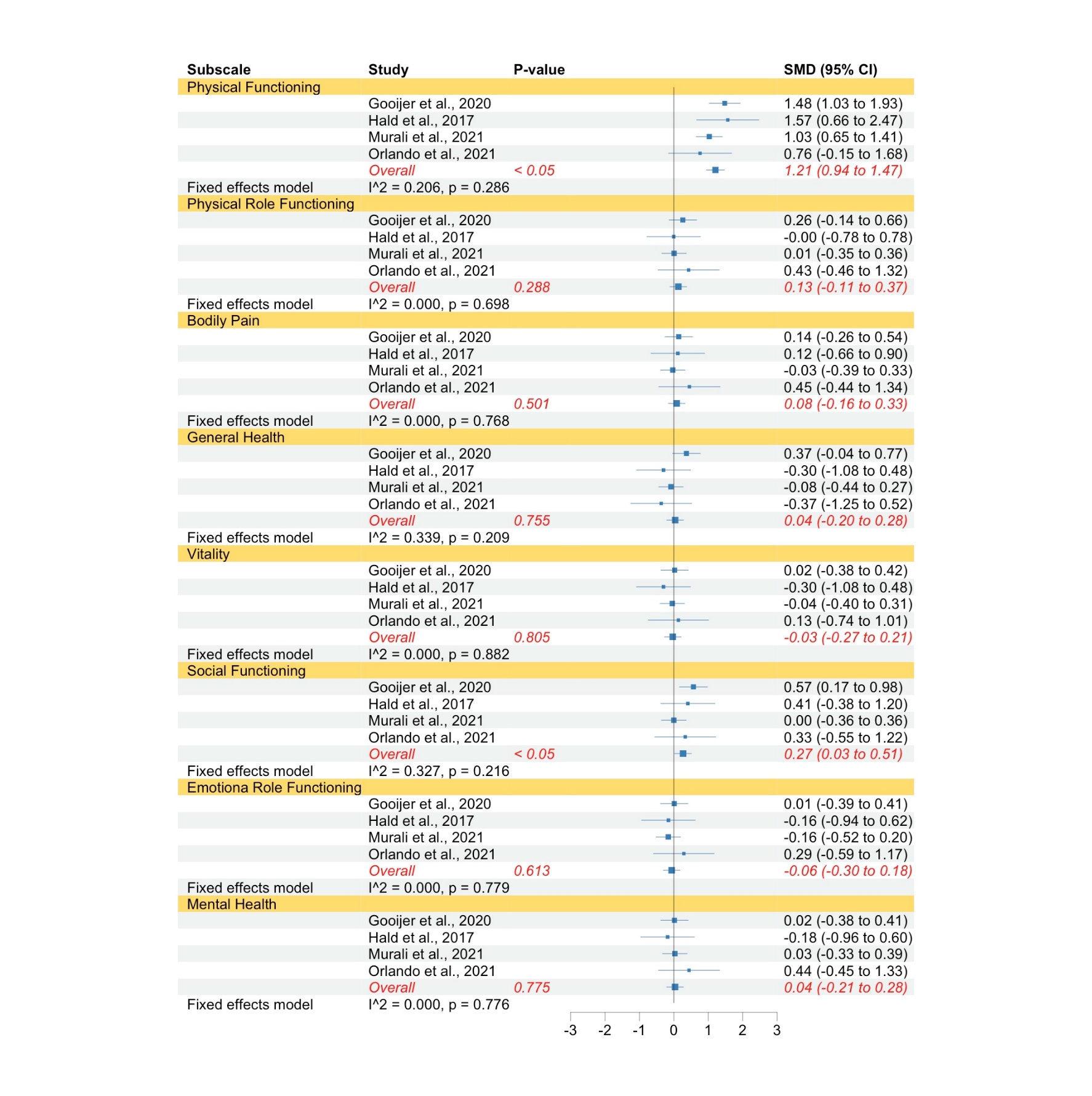


Abbreviations: SMD (Standardized Mean Difference).
