# Supplementary material for: Quality of life of pediatric and adult individuals with osteogenesis imperfecta: a meta-analysis
Source: Orphanet J Rare Dis. 2023 May 24;18:123. doi: 10.1186/s13023-023-02728-z (PMC10207627; doi:10.1186/s13023-023-02728-z)
Supplement: Supplementary file 5 — Additional file 5. Meta-regression of moderators of effects of QoL of adults with OI compared to norms. [file 13023_2023_2728_MOESM5_ESM.docx]

| **Additional File 5.** Meta-regression of moderators of effects of QoL of adults with OI compared to norms | | | | | | | |
| --- | --- | --- | --- | --- | --- | --- | --- |
| **Subscale** | **Moderator** | **N studies** | **β** | **95% CI** | | **P value** | **R^2^** |
|  |  |  |  | *lower* | *upper* |  |  |
| Physical functioning | | | | | | | |
| OI type I | | | | | | | |
|  | %females norm group | 3 | -.044 | -.145 | .056 | 388 | 0.00% |
|  | %females OI group | 3 | -.045 | -.077 | -.013 | < .01 | 87.29% |
|  | Age OI group | 3 | -.027 | -.182 | .129 | .739 | 0.00% |
| OI type III |  |  |  |  |  |  |  |
|  | %females norm group | 3 | -.068 | -.119 | -.017 | < .001 | 100.00% |
|  | %females OI group | 3 | -.014 | -.060 | -.033 | .567 | 0.00% |
|  | Age OI group | 3 | -.077 | -.138 | -.016 | < .05 | 100.00% |
| OI type IV |  |  |  |  |  |  |  |
|  | %females norm group | 3 | -.111 | -.317 | .095 | .291 | 3.94% |
|  | %females OI group | 3 | -.059 | -.097 | -.020 | < .001 | 86.83% |
|  | Age OI group | 3 | -.077 | -.415 | .262 | .657 | 0.00% |
| Physical role functioning | | | | | | | |
| OI type I | | | | | | | |
|  | %females norm group | 3 | -.135 | -.252 | -.018 | < .05 | 68.51% |
|  | %females OI group | 3 | -.001 | -.198 | .197 | .994 | 0.00% |
|  | Age OI group | 3 | -.181 | -.209 | -.152 | < .001 | 100.00% |
| OI type III |  |  |  |  |  |  |  |
|  | %females norm group | 3 | -.197 | -.247 | -.147 | < .001 | 100.00% |
|  | %females OI group | 3 | -.032 | -.160 | .096 | .625 | 0.00% |
|  | Age OI group | 3 | -.223 | -.400 | -.045 | < .05 | 74.57% |
| OI type IV |  |  |  |  |  |  |  |
|  | %females norm group | 3 | -.185 | -.276 | -.094 | < .001 | 90.83% |
|  | %females OI group | 3 | -.059 | -.160 | .043 | .256 | 14.36% |
|  | Age OI group | 3 | -.227 | -.280 | -.175 | < .001 | 100.00% |
| Bodily pain | | | | | | | |
| OI type I | | | | | | | |
|  | %females norm group | 3 | -.027 | -.052 | -.003 | .680 | 100.00% |
|  | %females OI group | 3 | -.012 | -.049 | .025 | .519 | 0.00% |
|  | Age OI group | 3 | -.028 | -.056 | -.000 | < .05 | 100.00% |
| OI type III |  |  |  |  |  |  |  |
|  | %females norm group | 3 | -.067 | .144 | -.158 | .144 | 47.03% |
|  | %females OI group | 3 | -.029 | -.063 | .005 | .099 | 60.78% |
|  | Age OI group | 3 | -.056 | -.227 | .114 | .516 | 0.00% |
| OI type IV |  |  |  |  |  |  |  |
|  | %females norm group | 3 | -.043 | -.088 | .002 | .062 | 100.00% |
|  | %females OI group | 3 | -.023 | -.045 | -.001 | < .05 | 100.00% |
|  | Age OI group | 3 | -.042 | -.137 | -.053 | .387 | 0.00% |
| General health | | | | | | | |
| OI type I | | | | | | | |
|  | %females norm group | 3 | -.023 | -.068 | .023 | .329 | 0.00% |
|  | %females OI group | 3 | -.022 | -.047 | .003 | .084 | 86.73% |
|  | Age OI group | 3 | -.016 | -.090 | .058 | .665 | 0.00% |
| OI type III |  |  |  |  |  |  |  |
|  | %females norm group | 3 | .010 | -.040 | .060 | .704 | 0.00% |
|  | %females OI group | 3 | -.009 | -.034 | .016 | .485 | 0.00% |
|  | Age OI group | 3 | .019 | -.041 | .079 | .528 | 0.00% |
| OI type IV |  |  |  |  |  |  |  |
|  | %females norm group | 3 | -.052 | -.097 | -.007 | < .05 | 100.00% |
|  | %females OI group | 3 | -.024 | -.046 | -.002 | < .05 | 100.00% |
|  | Age OI group | 3 | -.056 | -.109 | -.004 | < .05 | 100.00% |
| Vitality | | | | | | | |
| OI type I | | | | | | | |
|  | %females norm group | 3 | .024 | -.001 | .049 | .055 | 100.00% |
|  | %females OI group | 3 | .017 | -.015 | .049 | .295 | 0.00% |
|  | Age OI group | 3 | .024 | -.028 | .076 | .363 | 0.00% |
| OI type III |  |  |  |  |  |  |  |
|  | %females norm group | 3 | .008 | -.042 | .058 | .758 | 0.00% |
|  | %females OI group | 3 | .004 | -.021 | .028 | .779 | 0.00% |
|  | Age OI group | 3 | .008 | -.052 | .068 | .805 | 0.00% |
| OI type IV |  |  |  |  |  |  |  |
|  | %females norm group | 3 | -.003 | -.085 | .079 | .938 | 0.00% |
|  | %females OI group | 3 | .006 | -.028 | .040 | .723 | 0.00% |
|  | Age OI group | 3 | -.021 | -.106 | .065 | .633 | 0.00% |
| Social functioning | | | | | | | |
| OI type I | | | | | | | |
|  | %females norm group | 3 | -.009 | -.051 | .032 | .666 | 0.00% |
|  | %females OI group | 3 | -.018 | -.042 | .006 | .140 | 100.00% |
|  | Age OI group | 3 | -.002 | -.057 | .053 | .945 | 0.00% |
| OI type III |  |  |  |  |  |  |  |
|  | %females norm group | 3 | -.011 | -.061 | .039 | .674 | 0.00% |
|  | %females OI group | 3 | -.006 | -.031 | .019 | .631 | 0.00% |
|  | Age OI group | 3 | -.009 | -.069 | .051 | .760 | 0.00% |
| OI type IV |  |  |  |  |  |  |  |
|  | %females norm group | 3 | -.021 | -.129 | .087 | .703 | 0.00% |
|  | %females OI group | 3 | -.017 | -.051 | .018 | .336 | 0.00% |
|  | Age OI group | 3 | .000 | -.141 | .141 | .997 | 0.00% |
| Emotional role functioning | | | | | | | |
| OI type I | | | | | | | |
|  | %females norm group | 3 | -.095 | -.157 | -.034 | < .001 | 84.24% |
|  | %females OI group | 3 | -.048 | -.151 | .054 | .355 | 0.00% |
|  | Age OI group | 3 | -.095 | -.256 | .067 | .251 | 14.24% |
| OI type III |  |  |  |  |  |  |  |
|  | %females norm group | 3 | -.109 | -.172 | -.045 | < .001 | 93.84% |
|  | %females OI group | 3 | -.031 | -.095 | .032 | .329 | 0.00% |
|  | Age OI group | 3 | -.110 | -.294 | .075 | .244 | 18.31% |
| OI type IV |  |  |  |  |  |  |  |
|  | %females norm group | 3 | -.092 | -.192 | -.008 | .071 | 60.47% |
|  | %females OI group | 3 | -.024 | -.095 | .047 | .512 | 0.00% |
|  | Age OI group | 3 | -.126 | -.178 | -.074 | < .001 | 14.24% |
| Mental health | | | | | | | |
| OI type I | | | | | | | |
|  | %females norm group | 3 | -.021 | -.077 | .036 | .472 | 0.00% |
|  | %females OI group | 3 | -.025 | -.049 | -.001 | < .05 | 100.00% |
|  | Age OI group | 3 | -.011 | -.094 | .072 | .794 | 0.00% |
| OI type III |  |  |  |  |  |  |  |
|  | %females norm group | 3 | -.035 | -.126 | .056 | .448 | 0.00% |
|  | %females OI group | 3 | -.024 | -.049 | .001 | .061 | 100.00% |
|  | Age OI group | 3 | -.020 | -.158 | .118 | .777 | 0.00% |
| OI type IV |  |  |  |  |  |  |  |
|  | %females norm group | 3 | -.028 | -.976 | 3.274 | .231 | 0.00% |
|  | %females OI group | 3 | -.015 | -.037 | .007 | .179 | 0.00% |
|  | Age OI group | 3 | -.026 | -.078 | .026 | .326 | 0.00% |
| Abbreviations: CI (Confidence Interval). OI (Osteogenesis Imperfecta). | | | | | | | |
